# Applicant Fairness Perceptions of a Robot-Mediated Job Interview: A Video Vignette-Based Experimental Survey

**DOI:** 10.3389/frobt.2020.586263

**Published:** 2020-11-11

**Authors:** Sladjana Nørskov, Malene F. Damholdt, John P. Ulhøi, Morten B. Jensen, Charles Ess, Johanna Seibt

**Affiliations:** ^1^Department of Business Development and Technology, Aarhus University, Herning, Denmark; ^2^Department of Psychology and Behavioral Sciences, Aarhus University, Aarhus, Denmark; ^3^Department of Management, Aarhus University, Aarhus, Denmark; ^4^Department of Economics and Business Economics, Aarhus University, Aarhus, Denmark; ^5^Department of Media and Communication, University of Oslo, Oslo, Norway; ^6^Department of Philosophy and History of Ideas, Aarhus University, Aarhus, Denmark

**Keywords:** robot-mediated interview, fairness perceptions, implicit biases, fair proxy, job interview

## Abstract

It is well-established in the literature that biases (e. g., related to body size, ethnicity, race etc.) can occur during the employment interview and that applicants' fairness perceptions related to selection procedures can influence attitudes, intentions, and behaviors toward the recruiting organization. This study explores how social robotics may affect this situation. Using an online, video vignette-based experimental survey (*n* = 235), the study examines applicant fairness perceptions of two types of job interviews: a face-to-face and a robot-mediated interview. To reduce the risk of socially desirable responses, desensitize the topic, and detect any inconsistencies in the respondents' reactions to vignette scenarios, the study employs a first-person and a third-person perspective. In the robot-mediated interview, two teleoperated robots are used as fair proxies for the applicant and the interviewer, thus providing *symmetrical* visual anonymity unlike prior research that relied on asymmetrical anonymity, in which only one party was anonymized. This design is intended to eliminate visual cues that typically cause implicit biases and discrimination of applicants, but also to prevent biasing the interviewer's assessment through impression management tactics typically used by applicants. We hypothesize that fairness perception (i.e., procedural fairness and interactional fairness) and behavioral intentions (i.e., intentions of job acceptance, reapplication intentions, and recommendation intentions) will be higher in a robot-mediated job interview than in a face-to-face job interview, and that this effect will be stronger for introvert applicants. The study shows, contrary to our expectations, that the face-to-face interview is perceived as fairer, and that the applicant's personality (introvert vs. extravert) does not affect this perception. We discuss this finding and its implications, and address avenues for future research.

## Introduction

Personnel selection is one of the organizational activities that has experienced an increasing digitalization in the last few decades (Woods et al., 2020). Methods such as online applications (Sylva and Mol, 2009), digital interviews (Langer et al., 2017), and gamified assessments (Hawkes et al., 2018) have been found to provide practitioners with easier and faster selection procedures (Woods et al., 2020). While it has been argued that such digital selection procedures may be able to reduce implicit biases in applicant selection (Suen et al., 2019), research suggests that these methods may also replicate some of the biases from the traditional selection procedures (Lievens et al., 2015). To avoid replication of biases, recent research argues that the setup of the selection procedure may require change. In the context of job interview, Seibt and Vestergaard (2018) propose using a teleoperated robot as a fair proxy that removes visual cues (race, ethnicity, body size, etc.) that typically trigger implicit biases. This paper therefore empirically tests the fairness perceptions of using a robotic proxy in job interviews.

The employment interview is a critical organizational activity that helps organizations in recruiting the necessary workforce. It enjoys a widespread recognition among applicants and organizational decision-makers as one of the most commonly used selection techniques to assess candidates for employment (Macan, 2009). Significant executive resources and formalized efforts are allocated to identifying the “best” applicants by, for example, using well-documented tests based on scientific standards and evidence followed up by formal and (to varying degrees) structured interviews. Despite its long-held criticality in personnel selection, the employment interview has been found to be under the influence of implicit and potentially discriminating biases (Purkiss et al., 2006; García et al., 2008). Implicit biases involve rapid and automatic processing of information, which occurs unconsciously and tends to be difficult to control and change (Hinton, 2017). Holroyd (2012) provides an illustrative example of how implicit biases may work: “An individual harbors an implicit bias against some stigmatized group (G), when she has automatic cognitive or affective associations between (her concept of) G and some negative property (P) or stereotypic trait (T), which are accessible and can be operative in influencing judgment and behavior without the conscious awareness of the agent” (p. 275). For instance, in the case of employment interviews, implicit associations an interviewer may have related to physical appearance, obesity, race, and gender are some of the factors known to influence the way applicants are perceived and evaluated, thus resulting in the bias being manifested unintentionally (e.g., Heilman and Saruwatari, 1979; Johnson et al., 2010; Grant and Mizzy, 2014; Ruffle and Shtudiner, 2015). Allowing such biases into the selection process may reduce employee diversity and harm organizational reputation as well as organizational creativity and financial performance (Homan et al., 2007; Hewlett et al., 2013).

The subtle, unconscious effects of biases are not easy to deal with, and knowledge of how to effectively control and change biases is still incipient (Forscher and Devine, 2015). Extant research indicates that while implicit biases may be malleable, they tend to be highly resistant to change (Amodio, 2014). In a series of nine interventions, Lai et al. (2016) found that while the interventions were able to reduce implicit biases immediately, this effect was short-lived, i.e., it lasted between a few hours and a few days. Similarly, diversity training that is intended to make managers unbiased and inclusive has been shown to only have short-term effects (Dobbin and Kalev, 2016). It has further been argued that implicit biases are more likely to have a stronger effect during interactions between strangers than between acquaintances and friends (Landy, 2008). As the employment interview is most often an encounter between individuals who do not know each other, it is thus particularly exposed to implicit biases. In addition, interviewers commonly rely on intuition in their assessment and selection of applicants that may bias the selection decision. Even though the evidence suggests that intuitive selection is an inferior predictor of employee performance compared with analytical selection, research shows that HR professionals insist on relying on intuition in personnel selection, and in job interviews in particular (Highhouse, 2008).

As a response to the lack of effective means to control implicit biases in job interviews, we examine whether robots could be used to alleviate such biases. The goal is to test the potential of robots to increase objectivity and fairness in job interviews by using them as a *fair proxy communication* (FPC) device to “remove perceptual cues of implicit biases in order to increase the perceived fairness of decision-related communications” (Seibt and Vestergaard, 2018, p. 1). By eliminating some of the known triggers of implicit biases, such as visual cues that reveal physical appearance, age, ethnicity, gender, and even emotional states, a robotic proxy may hold the potential to create a fairer situation for applicants compared with a traditional face-to-face job interview (Nørskov and Ulhøi, 2020). While research shows that job interviews conducted via video conference or telephone are perceived as less fair than face-to-face interviews (Sears et al., 2013), the unique characteristics of a physically present robot may affect communication diferently when compared to a video conference and/or a telephone conference. Embodied and physically present agents in an interaction have indeed been found to be more engaging and capable of eliciting more favorable psychological responses (e.g., trust, empathy) and a greater sense of social presence compared to communication via a screen or a telephone (Li, 2015; Seo et al., 2015). If, in addition to these advantages, a teleoperated robot as a fair proxy is able to reduce or eliminate biases from the job interview, it seems reasonable to expect that a robot-mediated interview could yield higher perceptions of fairness than a face-to-face job interview. Our study thus examines whether a teleoperated robot could be used to mediate job interviews and increase applicants' fairness perceptions (i.e., procedural and interactional fairness) of the interview process and their behavioral intentions (i.e., intentions of job acceptance, reapplication intentions, and recommendation intentions), and whether applicant personality (introvert vs. extravert) affects this relationship. We investigate this question by employing an online vignette-based experimental survey from a first-person and a third-person perspective (for elaboration see section Procedures).

The paper makes two main contributions. Firstly, it tests a different conceptualization of FPC, than existing conceptual research has proposed, namely a *symmetrical* visual anonymity as opposed to the *asymmetrical* visual anonymity that was only recently conceptualized (Seibt and Vestergaard, 2018) and empirically tested (Adrian et al., 2019). When the robot-mediated job interview is based on the asymmetrical visual anonymity, it allows the applicant to see the interviewer via a computer screen, while the applicant is represented by a teleoperated robot. Such a setup seeks to create a fairer assessment situation for the applicant by removing visual cues related to the applicant's physical appearance, which is expected to limit the interviewer's biases toward the applicant. However, by also being able to see the interviewer and his/her nonverbal reactions, the applicant's may more easily engage in impression management tactics, which are known to bias the selection process and provide a false impression of the applicant (e.g., Cuddy et al., 2015). We therefore argue that the symmetrical visual anonymity—in which both parties are represented by a teleoperated robot—may serve to alleviate this risk and may thus be fairer to both parties. By reducing the opportunity for impression management, the symmetrical job interview places even more emphasis on the applicant's knowledge, abilities and skills as the objective criteria for applicant selection. Secondly, our study increases the understanding of when the robot-mediated job interview could be an effective alternative to the face-to-face job interview, including the possible target groups for this type of interview.

In the following, we present a review of the relevant literature and our hypotheses. We then explain our research design and report the results of the study. Finally, we discuss the findings and their implications.

## Theoretical Background and Hypotheses

Below we present the concepts of applicant fairness perceptions, behavioral intentions, Fair Proxy Communication (FPC), and applicant personality (introvert vs. extravert), and how they are interrelated. To understand how robot-mediation may affect the job interview process, we also discuss relevant literature on technology-mediation in job interviews and in communication allowing us to develop a set of hypotheses that the study tests.

### Applicant Fairness Perceptions and Behavioral Intentions in Job Interviews

Organizational justice deals with fairness perceptions in the workplace (Byrne and Cropanzano, 2001) and has its origins in social psychology research (Adams, 1965; Thibaut and Walker, 1975; Folger, 1977; Greenberg, 1987). Based on the organizational justice persepctive, Gilliland (1993) developed a model of applicants' reactions to selection systems. Research on applicant reactions investigates the “attitudes, affect, or cognitions an individual might have about the hiring process” (Ryan and Ployhart, 2000, p. 566). Our focus is on one particular type of applicant reactions, namely fairness perceptions of selection procedures. Gilliland's (1993) model was later taken up by Bauer et al. (2001) to develop the selection procedural justice scale for measuring applicant fairness perceptions of personnel selection. This scale is used to assess procedural, interactional and outcome fairness. While *procedural fairness* refers to the consistency and fairness of the applied job interview process and method, *interactional fairness* refers to the job interview being conducted in a respectful and informative way (Gilliland and Steiner, 1999). Finally, *outcome fairness* is related to the fairness of the decision that the job applicant receives from the hiring organization. In this study, we focus on applicant fairness perceptions of the job interview *setup*, and thus measure fairness only in terms of procedural and interactional fairness (Bauer et al., 2001).

Moreover, it is well-established that fairness perceptions of applicants have a positive effect on their behavioral intentions (Hausknecht et al., 2004; McLarty and Whitman, 2016). The latter construct refers to applicants' intentions to: (i) accept a job if offered one, (ii) recommend the organization to other jobseekers, and (iii) apply again for a job at the organization (Hausknecht et al., 2004; McLarty and Whitman, 2016). Behavioral intentions may thus affect an organization's ability to attract the best candidates (Ryan and Huth, 2008). The recommendation and reapplication intentions of candidates may significantly influence the quantity and the quality of prospective applicant pools (Kluger and Rothstein, 1993), which makes behavioral intentions important to predicting future behaviors of applicants.

### Implicit Biases in Job Interviews

Job interviews are particularly exposed to the risk of implicit biases because they typically involve face-to-face communication between strangers. Here, applicants' physical appearance, race, gender, etc. are observable and may influence how applicants are assessed. Indeed, extant research has documented that affective processes and subjective impressions prevail over applicants' qualifications and skills in job interviews (Graves and Powell, 1996; García et al., 2008; Huffcutt, 2011). It is well-established that automatic first reactions and the chemistry that emerges during the very first moments of two people meeting each other are sources of implicit biases (Zajonc, 1980; Howard and Ferris, 1996). Also, cultural similarity between an applicant and an employer increases the chances of the applicant being hired (Rivera, 2012, 2015). Similarly, applicants are more likely to be offered a job when the way they exhibit emotions (i.e., calm or excited) during job interviews is similar to the culturally-dependent expectations of interviewer (Bencharit et al., 2018). Additional sources of implicit biases are, for instance, the preference for interacting with people of the same gender (i.e., homosociality) and the tendency to have relations with people of similar sociodemographic background, personal and behavioral characteristics (i.e., homophily) (McPherson et al., 2001; Holgersson, 2013). Indeed, Rivera (2015) showed that homophily was the most dominant determinant in the assessment of applicants in job interviews. Furthermore, physical attractiveness also exposes applicants to implicit biases (Ruffle and Shtudiner, 2015). For example, when applying for non-managerial positions, physically attractive women have an advantage, while the opposite is the case for managerial positions (Heilman and Saruwatari, 1979). Allowing such biases to persist is likely to reduce diversity of employees, which may cause organizational performance and innovation as well as team creativity and team effectiveness to suffer (Homan et al., 2007; Hewlett et al., 2013). Designing fairer selection processes may thus benefit both applicants and organizations.

We test whether the robot-mediated interview may be able to eliminate visual cues and related biases and facilitate a situation where the interview procedure is more consistent and offers a more equal treatment of applicants than a face-to-face interview. Such a situation may help applicants focus on the contents of the interview and on demonstrating their knowledge and skills, giving them a better chance to perform (Gilliland, 1993). Indeed, Chapman and Rowe (2001) found that applicant competency ratings received a higher grading in videoconference-based interviews than in face-to-face interviews. The authors speculated that having a technology-based communication medium might have reduced applicant anxiety resulting in higher performance (Chapman and Rowe, 2001). Feelings of weak knees or sweaty palms are well-known reactions some applicants experience during a face-to-face employment interview. Anxiety has, for instance, been found to reduce hireability and job suitability ratings (Jeske et al., 2018). The employment interview has also been documented to be associated with applicant anxiety, which in turn affects the predictive validity of the employment interview and leads to the selection of less fitting applicants (McCarthy and Goffin, 2004). Similarly, shy individuals have been found to benefit from computer-mediated communication compared with face-to-face communication (Stritzke et al., 2004), which indicates a possible relevance of personality (introvert vs. extravert) for applicant perceptions of job interview methods. Technology-mediated communication may thus be useful to reduce or disguise nervousness and anxiety associated with face-to-face communication, especially for introvert personality types.

### Interaction Between the Two Main Effects

In the following three sections, i.e. Fair Proxy Communication in Job Interviews, Behavioral Intentions and Interview Setup, and Applicant Personality, Fairness Perceptions, Behavioral Intentions and Interview Setup, we treat the two main effects (robot-mediated vs. face-to-face interview and introvert vs. extravert personality) in relation to applicant fairness perceptions (measured as procedural and interactional fairness) and behavioral intentions. To justify that we treat the main effects separately, we first need to confirm H1. In order to proceed, we follow the recommendations in the literature regarding analysis of experimental designs (Kirk, 1995). A pre-requisite for handling the two main effects separately is that they operate independently of each other, i.e., that the effects of a robot-mediated vs. a face-to-face interview do not depend on whether we are looking at an introvert or an extravert applicant, or vice versa. Thus, we start by hypothesizing:

H1: The two main effects can be treated separately for all three constructs (procedural fairness, interactional fairness and behavioral intentions).

### Fair Proxy Communication in Job Interviews

**Figure 1:**
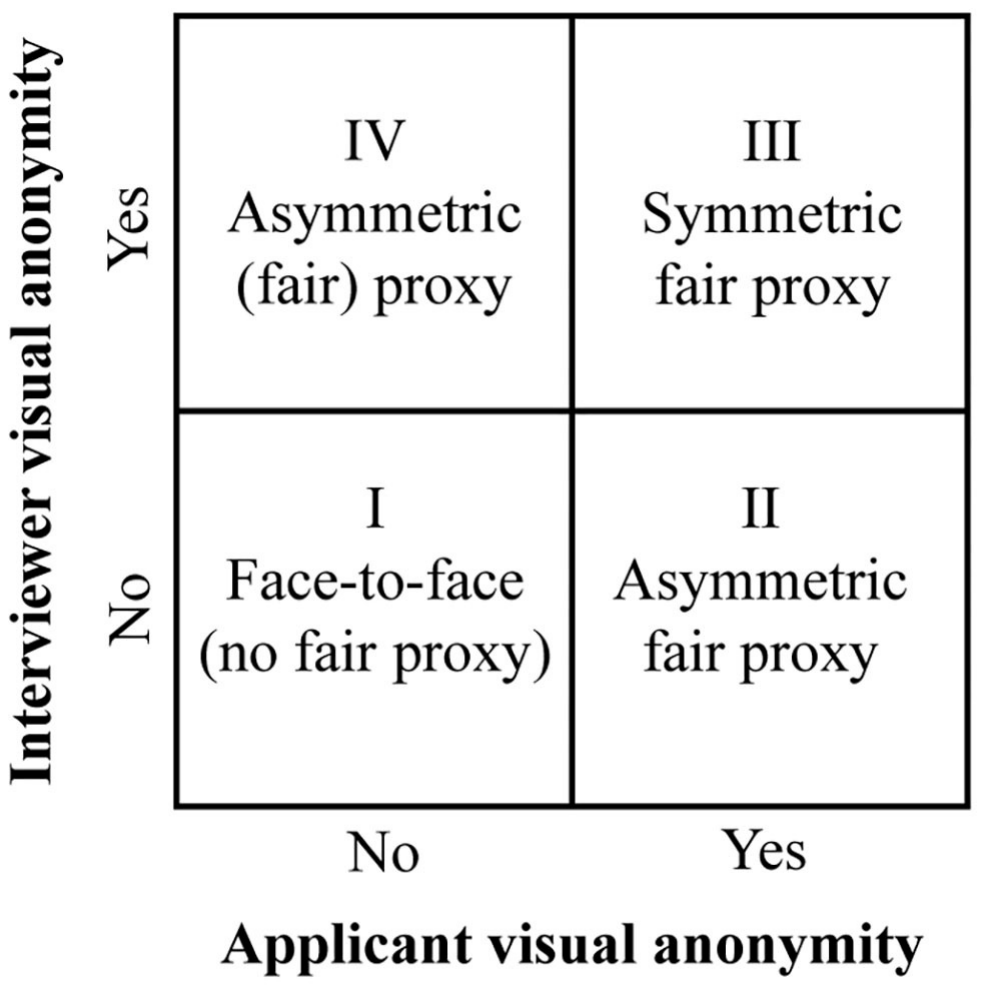
The four types of job interview setups with and without a fair proxy.

One way to secure a fairer applicant selection process is to use a robot as a fair proxy, an idea that is based on the principles of FPC (Seibt and Vestergaard, 2018). FPC provides a possible substitute for face-to-face communication and aims to improve the fairness perceptions of communication through the use of a robotic proxy. FPC thus pursues a practical-ethical objective of limiting the presence of perceptual cues that are known to trigger cognitive biases in face-to-face communication (Seibt and Vestergaard, 2018). Figure 1 shows the four types of job interviews and the roles FPC can play in this context. The starting point is the traditional face-to-face job interview without visual anonymity in quadrant I, while quadrant IV shows a situation in which only the interviewer is visually anonymous, because she is represented by a robotic proxy. This situation is deemed unfair to the applicant, as it places even more power into the hands of the interviewer by hiding her identity and non-verbal behavior, while allowing for potential perceptual biases toward the applicant. In between these two situations, quadrants II and III show the two interview setups that use FPC in a potentially beneficial way, i.e., to create more fairness in job interviews.

**Figure 2:**
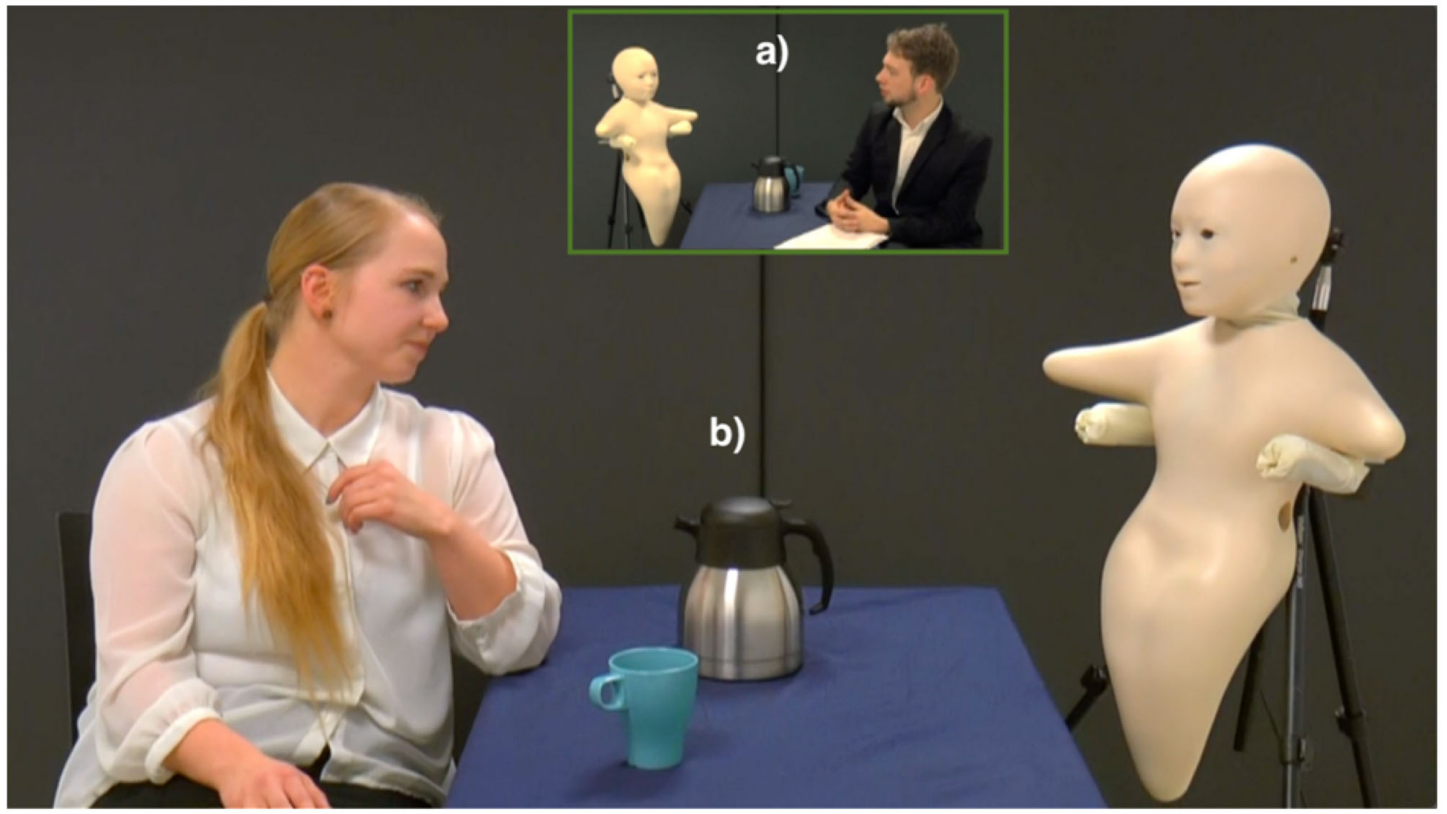
The “symmetrical” robot-mediated job interview, from the perspective of the interviewer **(a)** and the job candidate **(b)**. In **(a)**, a male interviewer communicates via the robot with a female job candidate, who is seated in another room, as shown in **(b)**. As the interviewer and the applicant communicate, their head movements, lip movements, and speech are transmitted via the robot.

The concept of FPC involves the use a fair proxy to provide an *asymmetrical* visual anonymity, as it uses a robotic proxy for only one communication partner. FPC is intended to create a fairer situation for the applicant, who is usually exposed to perceptual biases in face-to-face interviews (quadrant II). However, in this study we argue that a *symmetrica*l use of a fair proxy in job interviews may be equally relevant to consider (quadrant III). That is a situation in which the robot acts as a fair proxy for both the applicant and the interviewer (Figure 2). The reason for this is that applicants are known to engage in impression management techniques to improve their interview performance. Job interviews demand appropriate self-presentation to increase the applicants' chances of being hired (Kacmar et al., 1992; Proost et al., 2010).

Research suggests that interviewers should be cautious during applicant assessment, because self-presentation can be used in such a way that interviewers neglect competences and are swayed by the convincing self-presentation style of the applicant (Paulhus et al., 2013). As pointed out by Buzzanell (1999), prospective job applicants are typically advised to create the “right” image. A review of the literature has indicated that men and women engage in impression management tactics in business situations that tend to reflect stereotypical gender roles. Differently put, female organizational members tend to be stuck in a double bind where those who portray themselves as more communal and submissive are not chosen for leadership positions while candidates who try to utilize male tactics (such as being more aggressive) bear the negative consequences for violating normative gender roles (Guadagno and Cialdini, 2007). Studies have further shown that applicants can be trained to use non-verbal cues, such as body posture, to improve their chances of being hired. Cuddy et al. (2015) examined preparatory power posing, and found that adopting a high (vs. low) power pose in job interviews increases an applicant's chance of being hired. Such preparation and training for a job interview are used to manage impressions during the job interview and may additionally bias the selection process and provide a false impression of the applicant. We therefore examine a symmetrical FPC setting, which may serve to alleviate this risk.

Reducing visual cues in robot-mediated job interviews implies a certain degree of applicant anonymity. This is similar to telephone interviews, which have been found to change the applicants' and interviewers' style and content of communication (Harmon et al., 1995). For instance, Silvester and Anderson (2003) found that interviewers rated applicants higher in telephone interviews, when applicants attributed positive outcomes (of their prior employment) to personal causes, which was not the case in the face-to-face condition. Applicants were also found to use more personal attributions in telephone interviews compared to face-to-face interviews. The study also revealed a significant gender difference in face-to-face interviews that was not found in the telephone interviews. Male applicants were asked significantly more open questions during the face-to-face interviews and they talked more (i.e., produced more discourse). These findings suggest that visual anonymity in an interview shapes how the applicants present themselves, but also what information the interviewer will pay attention to, and consequently how the applicant will be assessed.

In their examination of text-based computer-mediated communication, Tidwell and Walther (2002) let dyads either meet face-to-face or communicate through email. They found that participants in the computer-mediated condition tried to overcome the limitations of such communication format by engaging in more personalized interactions. As a result, the participants got to know each other more efficiently with fewer message exchanges. This finding suggests that the lack of non-verbal cues in computer-mediated communication leads to a more intimate exchange compared to a face-to-face situation (Tidwell and Walther, 2002). Similarly, Joinson (2001) found that participants communicating through chat showed higher levels of spontaneous self-disclosure compared to those communicating face-to-face. Visually anonymous participants also disclosed more personal information than non-visually anonymous participants did. In both studies, the hyperpersonal model (Walther, 1996) was suggested as a possible explanation. Because feedback cues, and in particularly visual cues, are minimal in human-computer interaction, there is less information available to confirm or contradict our expectations. As a result, people choose to focus on cues that confirm their expectations, and may even adapt their behavior in order that make expectation confirmation more likely (Walther, 1996). So, although computer-mediated communication conveys less information about a conversation partner, it seems that this anonymity facilitates a more personal communication with a higher level of self-disclosure (Joinson, 2001; Tidwell and Walther, 2002).

With the aim to maintain anonymity while transmitting some of the facial information relevant for communication, Wang et al. (2014) examined the instant-messaging tool KinChat, which adds facial expression and head movement to text-based communication without revealing the user's face. The authors reported that the addition of facial expressions and head movement enhanced the level of understanding in communication (Wang et al., 2014). Robot-mediated interviews may therefore hold potential for promoting the information exchange in the job interview as well as reduce the effect of visual cues (e.g., gender) on the interviewers' way of communicating, thus limiting implicit biases.

Research on physically embodied robots showed that participants preferred interacting with an embodied robot than with a virtual one (Lee et al., 2006). Embodiment yields a greater sense of social presence, which was found to mediate the evaluation of the interaction. Wang and Rau (2019) also tested the effect of embodiment and found that embodied robots were preferred over other kinds of virtual reality, augmented reality, and telepresent robots. Kiesler et al. (2008) had similar findings. Their study compared an embodied humanoid robot and an on-screen robot. Both robots interviewed people about their health. Participants liked the embodied robot more, and attributed stronger and more positive personality traits to it, but they were more inhibited in their interaction. For instance, they would make fewer disclosures about socially undesirable behavior. The authors argued that the participants behaved as if they perceived the embodied robot as a human, and that this was not the case when communicating with the on-screen robot.

Papadopoulos et al. (2012) added a robot to a communication system in a collaborative computer game to enhance the remote communication of players. The presence of a robot increased the number of social cues (smiles and non-task-related speech) expressed by participants. Kim et al. (2009) investigated communication constraints in a human-robot interaction compared to human-human interaction. They found that human-human communication involved more social constraints (feelings, non-imposition, and disapproval), while task-oriented constraints (clarity and effectiveness) were applied equally for robot and human. Tanaka et al. (2014) compared video, avatar, and robot-mediated communication. They found that having a robot that was a physical embodiment of a communicator enhanced the social telepresence compared to video and audio-only conferencing. Research thus indicates that physically present robots may hold advantages for technology-mediated job interviews compared with on-screen robots, telephone, chat, etc. The main reason being that the feeling of social presence is greater, which has a positive impact on communication.

Indeed, Edwards et al. (2016) and Edwards et al. (2019) conducted a series of studies that investigated the expectations that participants have before interacting with a robot and how these change during and after their interaction. They documented that people anticipated less uncertainty, more liking, and more social presence when expecting to interact with a human compared to a robot. However, after a single brief interaction with a social robot, participants became less uncertain and felt more social presence than expected (Edwards et al., 2019). On the other hand, participants who had a brief interaction with another human seemed to lower their ratings of social presence after the interaction. The authors argued that the existence of hyperpersonal effects (Walther, 1996) in human-robot communication may explain the results. While face-to-face interactions entail more visual cues, which can contradict our expectations for the interaction and create less room for confirmation, in human-robot interaction people tend to focus on cues that confirm their expectations, which leads them to develop greater affinity for the other.

Extant research thus suggests that the use of different technologies in the employment interview has the potential to alter and improve the circumstances under which applicants are assessed by interviewers. Different technologies, among these embodied teleoperated robots, offer new perspectives on the employment interview, in particular by creating an environment with lower levels of perceptual cues for bias, which in turn promotes the information exchange in the job interview, facilitates increased personal communication with a higher level of self-disclosure, and involves fewer social constraints than face-to-face communication—all of which may contribute to increased fairness of applicant selection decisions. We therefore hypothesize:

H2: The average fairness perceptions (i.e., procedural fairness and interactional fairness) will be higher in a robot-mediated job interview than in a face-to-face job interview.

### Behavioral Intentions and Interview Setup

Applicant perceptions hold consequences for both organizations and applicants. Positive or negative applicant perceptions of the selection process may, for instance, affect applicants' work commitment, psychological well-being and performance, if the applicant accepts the job (Gilliland, 1993; Schuler, 1993; McCarthy et al., 2017). In general, applicant fairness perceptions are positively related to their behavioral intentions (i.e., intentions of job acceptance, reapplication intentions, and recommendation intentions) (McLarty and Whitman, 2016). A meta-analysis of 86 independent samples documented that applicants who had positive perceptions about the selection process were more likely to perceive the organization in a favorable light, and to exhibit stronger behavioral intentions (Hausknecht et al., 2004). This means that the perception of the selection process affects the hiring organization's reputation and its chances of attracting qualified workforce (Ryan and Huth, 2008). Thus, organizations are not the only party that is selecting. Applicants also select, e.g., whether they will apply, where they will apply, whether they will accept the job etc (Rynes, 1993). So, the higher the applicant perceptions, the higher will be their behavioral intentions will be (Nikolaou and Georgiou, 2018). We thus hypothesize:

H3: The average behavioral intentions will be higher in a robot-mediated job interview than in a face-to-face job interview.
H4: Fairness perceptions and behavioral intentions will be positively related.

### Applicant Personality, Fairness Perceptions, Behavioral Intentions and Interview Setup

Fairness perceptions and behavioral intentions of the interview may further vary depending on applicant personality. Extraversion is a core personality dimension, which is related to the social part of personality (Fishman et al., 2011). People who score high on introversion are typically associated with being reflective, shy and distant, while people who score high on extraversion are active, sociable and dominant (Gudjonsson et al., 2004). A study by Stritzke et al. (2004) found that shy individuals differed from non-shy individuals in their judgement of face-to-face situations, as measured by rejection sensitivity, initiating relationships, and self-disclosure. However, shy and non-shy individuals did not score differently on these measures in computer-mediated communication situations. The authors argued that the absence of visual and auditory cues in online environments makes detecting negative or inhibitory feedback cues from others less likely, and improves the communication experience of shy individuals. Similarly, Hammick and Lee (2014) found that shy individuals felt less communication apprehension (i.e., fear or anxiety that something bad will happen in the communication) in virtual reality compared to a real-life face-to-face interaction. Robot-mediated interviews may therefore be able to create a more comfortable situation for those with introvert personality type.

Since extraverts process social stimuli differently than introverts, and since they enjoy social interactions more than introverts (Fishman et al., 2011), the two personality types may thus prefer different types of job interviews, which is expected to influence their fairness perceptions and behavioral intentions. Research suggests that a favorability bias toward extraverts exists in Western societies (Andersen and Klatzky, 1987; Paulhus and Morgan, 1997; Swann and Rentfrow, 2001), which may put introvert applicants at a disadvantage during job interviews. Unlike extraverts, introverts are more likely to experience social interactions as overstimulating and draining (Keirsey, 1998). Removing some of the visual cues involved in job interviews may reduce social stimuli to a degree that makes the situation more comfortable for introvert applicants, thus allowing them to perform better during a job interview. We therefore hypothesize the following:

H5: Compared with extravert applicants, introvert applicants' fairness perceptions will be higher in a robot-mediated job interview.
H6: Compared with extravert applicants, introvert applicants' behavioral intentions will be higher in a robot-mediated job interview.

## Research Design

The present study was based on an online experimental survey with video vignettes. A vignette can be defined as a carefully prepared description of a person, object or situation, representing a systematic combination of properties (Atzmüller and Steiner, 2010, p. 128). Vignettes have been acknowledged for being especially valuable in exploring perceptions, attitudes and behaviors, and for not necessarily requiring participants to have in-depth knowledge of the research topic in questions (Hughes, 2008, p. 918).

The use of experimental vignette method (EVM) in business studies can be found in, for example, investigations of accounting environments regarding ethical decision situations, with accountants and accounting students as participants (Smith and Rogers, 2000). EVM has also been applied to investigate how the ways decision explanations are presented may influence respondents' (graduate students') perceptions of trustworthiness (Elsbach and Elofson, 2000). The use of EVM, however, is not restricted to surveying students, but also to evidence real-life managers' ethical behaviors when their own economic well-being may be at stake (Hoffman et al., 1998). In the study of discrimination in job interviews, “fictitious” setups (such as experimental vignettes) that use students as participants have also surfaced (e.g., Kutcher and Bragger, 2004; Krings and Olivares, 2007; Lindner et al., 2014; Gioaba and Krings, 2017).

Notwithstanding, as emphasized by Aguinis and Bradley (2014), EVM need not be limited to a written format. Rather, EVM can include images, videos and other media. More recently, the application of scripted video vignettes has emerged. Such vignettes represent short, visual depictions of pre-written (hypothetical) events (Hillen et al., 2013, p. 296). This vignette format has increased realism (Burt et al., 2016) and engagement (Davies et al., 2016). Nonetheless, this kind of EVM is still a methodology in progress and as cautioned by Hillen et al. (2013) from their review on applying scripted video vignettes for experimental physician-patient communication research: “No “gold standard” exists for most methodological issues encountered when conducting this type of research, as literature testing the consequences of different approaches is lacking” (p. 308).

However, considering the mentioned advantages of video vignettes, we conducted an online experimental video-vignette-based survey, which employed a 2 (personality type, introvert or extravert) by 2 (type of job interview, face-to-face or robot-mediated) experimental survey design. As the technology is relatively new in this context, we found it critical to ensure that the context was clearly presented in the survey. To ensure that the respondents all had the same understanding of the two job interview situations (robot-mediated vs. face-to-face) before responding to the survey questions, the survey included a link to a scripted video displaying a job applicant attending a job interview (with and without a fair proxy involved). Since the study examines four different conditions (2 × 2 design), each condition was shown in a separate video. Survey respondents were randomly assigned to two of the four conditions. Randomization was undertaken automatically by the Qualtrics survey software.

### Method

#### Participants

Study 1 was designed to test H1–H3. In this study, we conducted a web-based experimental survey among bachelor students of business administration at a Danish university. Respondents were contacted through the University mailing list. We received 235 valid responses to the survey. Student respondents were selected because we expected these respondents to possess specific characteristics. First, we expected this age group to be more used to adopting new technologies. Second, given their limited work experiences and future high-in-demand attractiveness in the present war-for-talent business situation, this group is less likely to expect to be discriminated against in the selection process.

#### Procedures

The experimental survey was delivered through the online survey platform Qualtrics. On the first page of the survey the respondents were informed of the overall purpose of the study and were asked to give their consent to participate. A total of 279 respondents took part in the survey, whereof 44 were excluded due to incomplete data (≥50% missing items) leaving 235 valid responses. Hereof, 109 (67 male, mean age 22.8 years) respondents were randomly assigned to watch two videos (a face-to-face job interview and a robot-mediated job interview), in which the applicant was an introvert. The remaining 126 (81 male, 45 female, mean age 22.9 years) respondents were randomly assigned to watch two videos (a face-to-face job interview and a robot-mediated job interview), in which the applicant was an extravert. The videos were shown in a random order whereby some respondents were first exposed to the face-to-face interview and others to the robot-mediated interview. Before and after each exposure to the video stimuli the respondents were asked to fill in questionnaires (see below). The videos were introduced with the following introduction to the videos:

“In the following, you will see two short video segments that we ask you to rate on different parameters. Both videos portray an interview between Anna and Mark. Anna is a job applicant, and Mark is a recruiter for an international company. Anna is a young product designer who graduated from the School of Design in Copenhagen 4 years ago. Anna was employed immediately after graduation and worked for the company “D.S. Design” in Copenhagen for nearly 4 years. She gained increasing responsibility and was the lead designer on several products. Anna loved the creative environment in D.S. Design but there were not many opportunities for advancement. Furthermore, 4 months ago she had to move to Aarhus for personal reasons. She has been unemployed ever since, but has now applied for a position in Alpha Designs, an international design company based in Aarhus. Alpha Designs has invited Anna for an interview, since her profile is a perfect match to the position they seek to fill. The two short videos presented in this survey show Anna being interviewed by Mark, a recruiter from Alpha Designs. The two videos show two different job interview situations. One is a face-to-face job interview, while the other one is robot-mediated.”

In this study, we sought to uncover how respondents react to the video vignette scenarios from a first-person and a third-person perspective. We did this for the following reasons. Firstly, the third-person perspective on vignette scenarios may reduce the social desirability of responses (Wason et al., 2002; Hughes and Huby, 2004) and desensitize possibly sensitive or controversial topics (Finch, 1987). Social robots have indeed been shown to pose significant ethical and moral challenges (Van Wynsberghe, 2013; Vallor, 2015). In addition, social robots in job interviews are not yet a part of a personnel selection practices in organizations. As such, the investigated concept of the robot-mediated interview is novel and may be perceived as unusual or even eccentric. The use of vignettes, where participants cannot be expected to have previous experiences with the technology, can therefore help provide focus for the participants and clarify the principles under study (Hughes, 2008, p. 920). It has been argued that the paradoxical blend of animate and inanimate features of social robotic agents escalates the novelty experience during interactions with such agents (Smedegaard, 2019). All of this may potentially make the robot-mediated interview a sensitive concept, which is why combining a third-person and a first-person perspective is necessary.

Secondly, research using vignettes has indeed documented a discrepancy between what respondents think should happen and what they do themselves (Carlson, 1996). We therefore made use of both a first-person and a third-person perspective to tap into the two dependent variables: perceptions of applicant fairness and behavioral intentions. More specifically, applicant fairness perceptions were assessed from a third-person perspective, and behavioral intentions from a first-person perspective. In the case of the latter, the respondents were asked to respond, as if they were the applicant in the video (e.g., “If I were the applicant in the video, I would accept the job if it was offered to me”). Because it is well-established in the personnel selection literature that applicant fairness perceptions and behavioral intentions are positively related (e.g., McLarty and Whitman, 2016), investigating the two variables from two different perspectives was expected to detect any differences and/or contradictions in the respondents' reactions to vignette scenarios, and increase the validity of the results. So, if fairness perceptions and behavioral intentions are positively related in the robot-mediated condition, even though they are assessed from two different perspectives, this may indicate that the responses given did not involve social desirability effects and/or that the vignette scenarios were not perceived as a sensitive topic.

Finally, at the end of the survey, we also included an open-ended question to capture possible explanations of our findings and reveal socially situated aspects of respondents' responses.

#### Measures

We used well-validated scales for each of the constructs. Procedural and interactional fairness were measured using Bauer et al. (2001) scale. To assess behavioral intentions, we relied on McLarty and Whitman (2016) scale. Table 1 includes the wording for all items. The goal of the analysis is to determine the existence of differences in the means for the three constructs based on the experimental condition. From the description of the experiment, this is a classical 2 × 2 within-between design.

**Table 1:** Items used for measuring the three constructs.

**Item**	**Wording**
Procedural fairness item 1	I think that using this interview process was a neutral and unbiased way to select people for the job of product designer.
Procedural fairness item 2	I think that the interview questions themselves were fair.
Procedural fairness item 3	Overall, the method of interviewing used was fair.
Interactional fairness item 1	Has the interviewer treated the applicant in a polite manner?
Interactional fairness item 2	Has the interviewer treated the applicant with dignity?
Interactional fairness item 3	Has the interviewer treated the applicant with respect?
Interactional fairness item 4	Has the interviewer refrained from improper remarks or comments?
Behavioral intentions item 1	I would accept the job if it was offered to me.
Behavioral intentions item 2	I would apply to this organization again.
Behavioral intentions item 3	Based on my experience with this interview process, I would encourage others to apply for employment with this organization.

#### Video Stimuli

**Figure 3:**
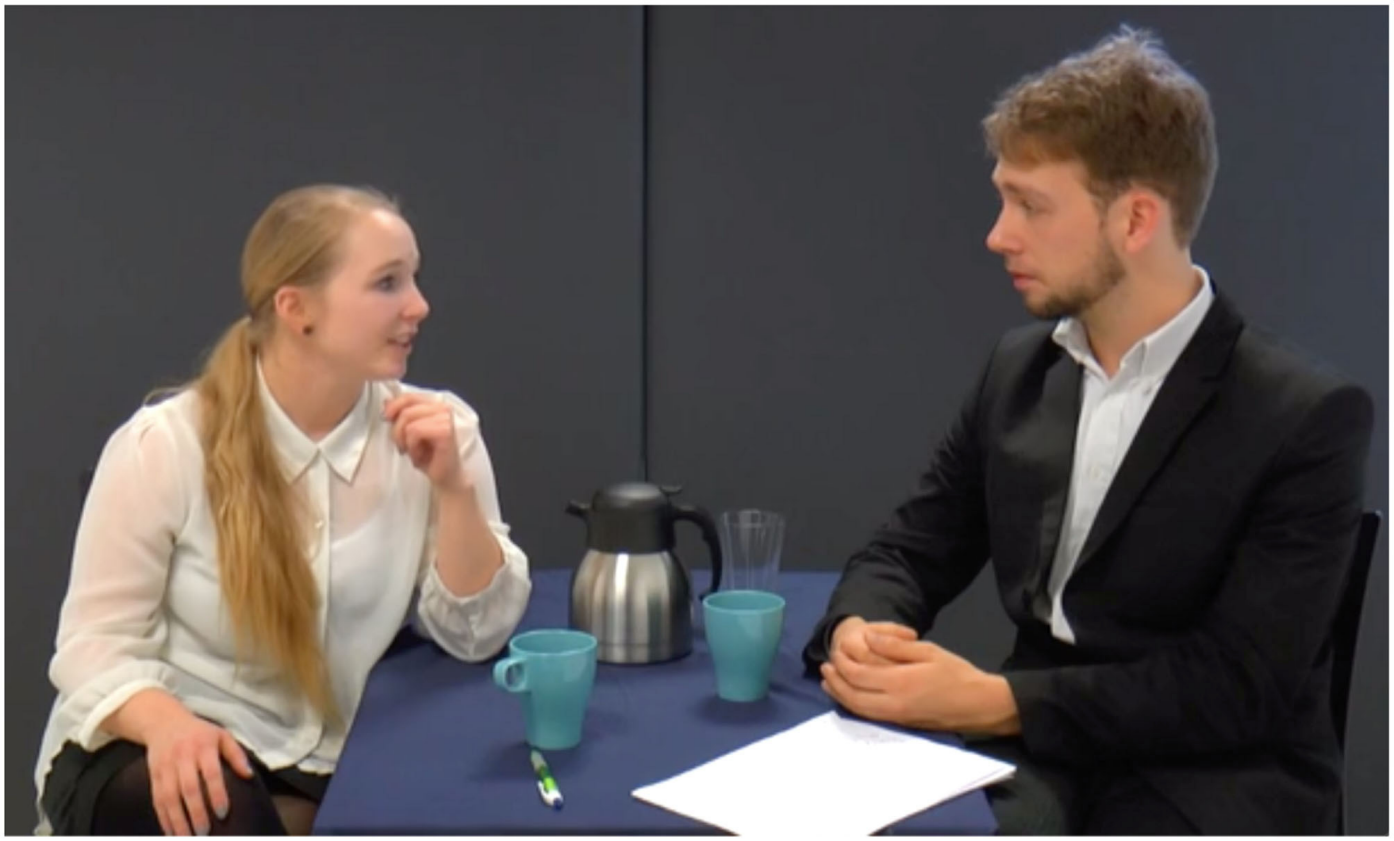
A scene from the face-to-face job interview that was used in the survey.

The four scripted videos, each lasting ~40 s, and each representing the four conditions, displayed a female job applicant attending a job interview (with and without a fair proxy involved). The applicant was interviewed by a male interviewee, and the interview dialogue was identical in all four videos. Figure 2 shows the image from the video that included a fair proxy, while Figure 3 shows the face-to-face job interview. In the robot-mediated setup in Figure 2, the applicant and the interviewer are seated in two different rooms, each of them sitting with the robotic proxy representing the other party. Each robot is teleoperated by the party it represents, and neither party can see the other. In the videos showing a face-to-face job interview, the applicant and the interviewee were seated in the same room.

#### The Robot

We used a teleoperated android robot, Telenoid R1, developed by the Japanese robotics lab ATR Hiroshi Ishiguro Laboratories. In the videos, the Telenoid mimicked the operator's head movements, emulated the operator's lip movements, and transmitted the operator's speech. Visually, it is designed to display a minimal human embodiment (Ishiguro, 2016), appearing “both old and young” and “both male and female” (Seibt and Vestergaard, 2018, p. 9). Extant studies have suggested that the robot is perceived as lacking social identities (e.g., age, gender) and visual cues, which made it easier for people to focus on the conversation (Seibt and Vestergaard, 2018). For these reasons, the Telenoid was selected to be studied in the job interview setting.

### Analysis

To examine the extent to which differences in means between the various experimental conditions exist, we use SMM (Structured Mean Model) analysis presented in Breitsohl (2019). This analytical approach is also known as MACS (Mean and Covariance Structures) analysis (see Bagozzi, 1977; Cole et al., 1993; Ployhart and Oswald, 2004) and is closely related to the seminal paper by Meredith (1993) addressing measurement invariance. Among its key merits compared to traditional ANOVA analysis are the ability to consider measurement error (as opposed to simply calculating an average for a specific scale) as well as handling missing values in an integrated way (although missing values are not a problem for our sample). Disregarding measurement errors may lead to an attenuation in sensitivity to detect specific population mean differences as described by Cohen (1988, p. 536).

**Figure 4:**
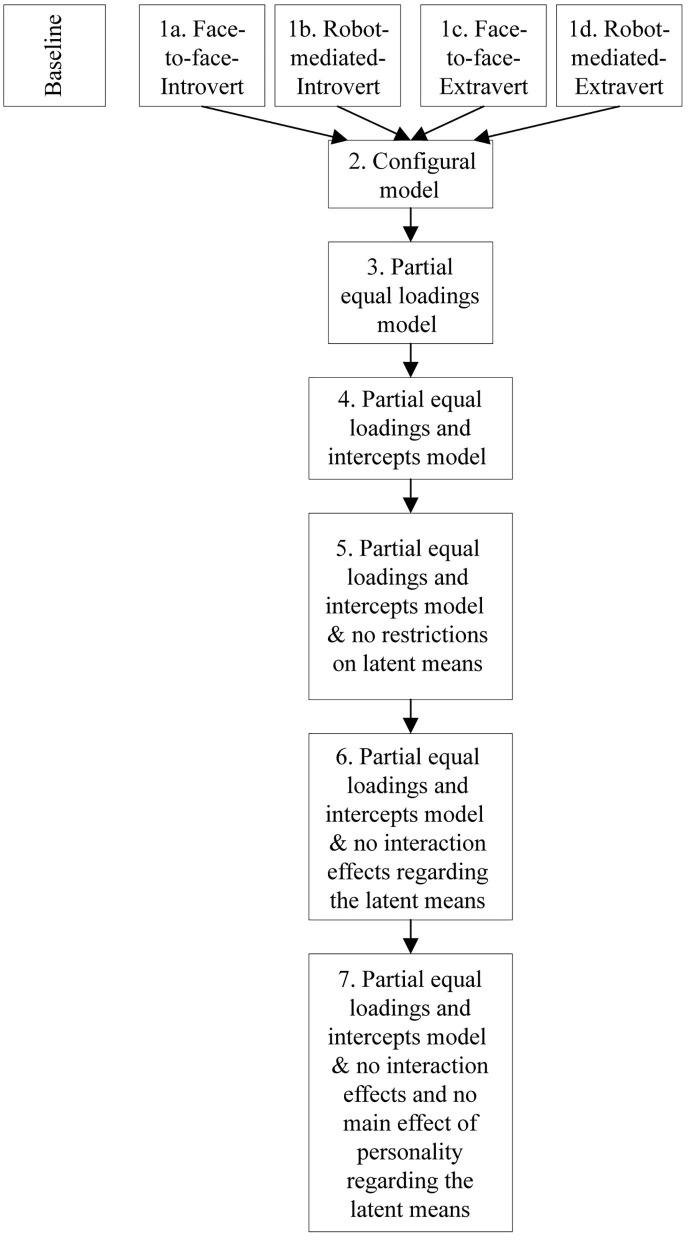
Overview of the SMM framework.

We follow a sequential process in carrying out SMM as suggested in Byrne (2012). This involves estimating, assessing and testing a sequence of increasingly restricted confirmatory factor analysis (CFA) models. In line with the existing literature concerning CFA models, we use CFI (Bentler, 1990) and TLI (Tucker and Lewis, 1973) as measures of goodness-of-fit with a threshold of around 0.95 signifying well-fitting models for both. We also assess Root Mean Square Error of Approximation (RMSEA) suggested by Steiger and Lind (1980) (cf. Steiger, 2016) with values below 0.05 signifying good fit, values between 0.05 and 0.08 signifying reasonable error of approximation, and values between 0.08 and 0.1 associated with mediocre fit. Finally, Standardized Root Mean Square Residual (SRMR) values <0.08 signify good fit. Each of the three scales employs a five-point Likert scale. To accommodate obvious deviations from normality we used the MLM estimator, which is a robust alternative to the usual ML estimator associated with confirmatory factor analysis. Hence, to assess the validity of the increasingly restricted sequence of models we use the scaled difference chi-square test (Δ MLM) (Satorra and Bentler, 2001). All analyses are carried out using Mplus 7.11 (Muthén and Muthén, 2012). Figure 4 provides an overview of the SMM framework. Steps 1a-1d in the abovementioned sequence entail establishing well-fitting baseline models. We do this by estimating for each of the four conditions and scrutinizing the adequacy of each model. Step 2 is to estimate a well-fitting configural model. In this setup, the configural model equals the model obtained by jointly estimating all four baseline models. Given that the baseline models in Steps 1a-1d are well-fitting the configural model will typically also be well-fitting.

Step 3 addresses the question of equal loadings of the CFA model across the four experimental conditions. We carry out this assessment using the configural model from step 2 as the point of departure. In an iterative procedure, we assess whether equality restrictions regarding the loadings across the four conditions for every construct are warranted using the scaled difference chi-square test. In a similar vein, we assess whether equality restrictions regarding the indicator intercepts are justifiable in step 4. Thus, having established partial measurement invariance, we can finally test for differences across the four groups in terms of means of the latent variables. In step 5, we set the basis for the analyses of the structural part by estimating a model with the same set of restrictions regarding the measurement part as was the result of step 4 and no equality restrictions at all regarding the latent means across the four conditions. Step 6 imposes restrictions regarding the latent means corresponding to no interaction effects. Finally, step 7, based on a non-significant increase in the scaled chi-square test between step 5 and step 6, assesses the viability of restrictions corresponding to no main effects. The models from step 5 to 7 are used to test all our hypotheses. We analyze differences in latent means based on a structural model where all three constructs are allowed to correlate as well as a structural model, where behavioral intentions is the dependent variable, and procedural fairness and behavioral fairness act as covariates.

### Results

As suggested by Brown (2015), in situations where the indicators are expected to be correlated due to temporal dependence (the same construct being measured on two different occasions) it is common to permit for this kind of dependence by allowing the error terms in a CFA model to be correlated. However, when we allowed for temporal dependence (the within-subject part of the design) in a preliminary analysis, none of the correlations between the error terms were significant. This may not be too surprising given that we do not have a genuine longitudinal design, but merely a situation where the same subjects provide answers to two different situations (robot-mediated vs. face-to-face). Thus, allowing for correlation between the constructs driving the manifest indicators leaves no residual correlation between those indicators' error terms. Hence, we analyze the data from this experiment as a 2 × 2 between-subjects design. This is also in accordance with the recommendations in Ployhart and Oswald (2004).

The first step established baseline models for each of the four experimental conditions (face-to-face introvert, face-to-face extravert, robot-mediated introvert, and robot-mediated extravert). Table 2 shows that all baseline models satisfy the requirements outlined in the methods section. A possible exception might be RMSEA for robot-mediated-introvert. However, the RMSEA is just above the threshold for mediocre fit and the remaining goodness-of-indices all point at a well-fitting baseline model.

**Table 2:** Results of SMM analysis.

	**RMSEA**	**CFI**	**TLI**	**SRMR**	**d.f**.	**Δ MLM**
Baseline: Face-to-face-introvert	0.000	1.000	1.003	0.044	32	
Baseline: Face-to-face-extravert	0.063	0.969	0.957	0.049	32	
Baseline: Robot-mediated-introvert	0.088	0.939	0.915	0.071	32	
Baseline: Robot-mediated-extravert	0.061	0.966	0.953	0.070	32	
Configural model	0.054	0.979	0.970	0.060	128	
Partial equal loadings model	0.053	0.975	0.971	0.120	155	33.96
Partial equal loadings and intercepts model	0.073	0.947	0.947	0.153	182	98.79^**^
No restrictions on latent means	0.060	0.967	0.964	0.071	164	
No interaction effects for any of the latent means	0.059	0.967	0.965	0.071	167	1.188
No interactions and no effect of personality for any of the latent means	0.058	0.968	0.966	0.073	170	1.898

* *p-value < 0.05*,

** *p-value < 0.01*.

*The Δ MLM statistic tests the model in the current line against the model in the previous line*.

Next followed the estimation of a configural model, which is a single model comprising all four baseline models without any limitations on the estimated parameters (except for those relevant for identification). As this corresponds to the simultaneous estimation of the four baseline models, it can be established that the configural model is a well-fitting model.

Third, we addressed the question of equal factor loadings. The model reported in Table 2 is an equal factor-loading model, where all factor loadings are equal except for item 2 for procedural fairness, which can operate freely across the four conditions.

During the fourth step, we imposed restrictions in terms of equal indicator means. The model reported in Table 2 has equal indicator means for all indicators except for item 3 for procedural fairness, item 2 for behavioral intentions, and item 3 for behavioral intentions. For the latter three indicators the means are constrained to be equal for robot-mediated extravert and robot-mediated introvert combinations as well as for the face-to-face introvert and face-to-face extravert combinations. Although the necessary restrictions are not justifiable from a statistical point of view (*p*-value < 0.001), the final model does constitute a well-fitting model based on the criteria outlined.

This allowed us to examine the structural part of the CFA model, which is the means and variances of the latent variables. Of particular relevance for this explorative study is the extent to which the latent means differ across the four experimental conditions. Table 3 holds the estimated means of the latent variables for the four conditions in the model without any restrictions on the latent means (step 5 in the SMM procedure). We move ahead in a stepwise fashion as recommended in the literature (see e.g., Kirk, 1995). Based on the non-significant scaled chi-square test between the models from step 5 and step 6, our first intermediate result states that there are no interaction effects of personality of applicant and type of interview for any of the means of the three latent variables. Thus, we cannot reject H1 (see Table 2). Furthermore, based on the non-significant scaled chi-square test between the models from step 6 and step 7, H5 and H6 are rejected, i.e., there is no effect of the personality of the applicant for any of the latent means. Table 4 holds the standardized estimated parameter estimates of the difference in means between the face-to-face and the robot-mediated interviews for each of the three latent variables. Based on the results in Table 4, we reject H2 and H3, i.e., the means of the latent variables depend on the type of interview but the direction is opposite to what we expected. Table 5 shows the parameter estimates for the latent means in a model where behavioral intentions are driven by the two fairness constructs. Interestingly, the parameter estimate for the mean of behavioral intentions is somewhat smaller compared to the unrestricted model (−0.488 compared to −0.711). However, the parameter estimate is still statistically significant. Thus, even when we control for the differences in means between the face-to-face and the robot-mediated interviews for fairness perceptions, there is still a difference between the means for behavioral intentions. Finally, Table 6 shows the associated slope coefficients from the model reported in Table 5. We have a positive and significant relationship between behavioral intentions and procedural as well as interactional fairness, thus confirming H4.

**Table 3:** Parameter estimates of latent means.

	**Mean of procedural fairness**	**Mean of interactional fairness**	**Mean of behavioral intentions**
Face-to-face-introvert (reference group)	0 (0)	0 (0)	0 (0)
Robot-mediated-introvert	−0.398 (0.219)	−0.443 (0.141)	−0.831 (0.206)
Face-to-face-extravert	0.077 (0.138)	0.049 (0.121)	0.226 (0.135)
Robot-mediated-extravert	−0.325 (0.203)	−0.405 (0.144)	−0.823 (0.201)

*Standardized estimates (standard error). Initial latent model*.

**Table 4:** Parameter estimates of latent means.

	**Mean of procedural fairness**	**Mean of interactional fairness**	**Mean of behavioral intentions**
Face-to-face (reference group)	0 (0)	0 (0)	0 (0)
Robot-mediated	−0.287 (0.117)	−0.438 (0.098)	−0.711 (0.115)

*Standardized estimates (standard error). Final latent model*.

**Table 5:** Parameter estimates of latent means.

	**Mean of procedural fairness**	**Mean of interactional fairness**	**Mean of behavioral intentions**
Face-to-face (reference group)	0 (0)	0 (0)	0 (0)
Robot-mediated	−0.293 (0.119)	−0.445 (0.098)	−0.488 (0.108)

*Standardized estimates (standard error). Controlling for covariates*.

**Table 6:** Parameter estimates of the relationship between behavioral intentions and fairness.

	**Behavioral intentions**	
	**Robot-mediated**	**Face-to-face**
Procedural fairness	0.326(0.085)	0.400 (0.078)
Interactional fairness	0.442 (0.080)	0.223 (0.061)

*Standardized estimates (standard error)*.

Finally, since only 11 respondents provided answers to the open-ended question, it did not justify an elaborate analysis of these data. Nonetheless, we categorized the answers into themes that indicate some potential explanations for our survey findings. The answers were related to: (i) the perceptions of and attitudes toward the robot-mediated job interview, and (ii) perceptions of the robot's physical appearance. The answers did not reflect a clear-cut attitude toward the concept of robots as proxies in job interviews, but pointed out both positive and negative aspects of the concept. Some expressed a general, positive perception of the concept, i.e., “wonderful robotic interview”, “I find this robot really impressive idea,” “the idea behind this technology […] is brilliant.” Others had negative perceptions, and in particular pointed out three interrelated aspects, which may get lost in the robot-mediated job interview: *emotions* that do not get transmitted due to the robot's design, the loss of *intimacy* between the interviewer and the applicant, and the lack of opportunity to feel the *chemistry* between the two parties (e.g., “I will always prefer a face-to-face interview, so they can get a feel of me and I can get a feel of them”). Nonetheless, one respondent remarked that “the technology works fine if it is impossible to do the interview in person,” indicating that the robot-mediated interview could be appropriate in certain situations, but should not replace the face-to-face interview completely. Finally, the appearance of the robot was perceived negatively by some, e.g., “[it is] somehow in a crucified posture,” and “[the interview becomes] bizarre because of the doll.” While we cannot generalize based on the few responses generated by the open-ended question, these responses may indicate some of the reasons for our finding that the face-to-face interview is perceived more fairly, namely the robot's physical appearance and the affective dimensions of interpersonal communication that are removed from the robot-mediate setup. The responses also indicate that the concept in itself has potential, but that another type of robot could be more suitable.

## Discussion

This paper addresses a novel area of inquiry, namely the robot-mediated job interview. The paper makes two main contributions. Firstly, it examines the case of a symmetrical visual anonymity in a job interview as opposed to the asymmetrical visual anonymity that prior research has conceptualized (Seibt and Vestergaard, 2018). Secondly, it contributes to the understanding of when the robot-mediated job interview may be a suitable alternative to the face-to-face job interview.

Our main finding showed that face-to-face employment interviews were perceived as fairer. This was the case both in the condition involving an introvert applicant and in the condition involving an extravert applicant. This lack of effect of applicant personality is somewhat surprising, as extant research has documented positive effects of impression management in job interviews, thus favoring extravert applicants. The perception that face-to-face interview is fairer is also unexpected considering that face-to-face communication is particularly conducive to discrimination (Rivera, 2012). We further found that applicants' fairness perceptions positively affect their behavioral intentions. The intentions to accept the job (if offered one), reapply to the organization, and recommend it to other jobseekers were thus higher in the face-to-face setup than in the robot-mediated setup. However, when controlling for fairness perception of the two setups, the difference in behavioral intentions between the setups was still present and significant, albeit the effect was not as strong. This finding indicates that other factors than fairness perceptions need to be considered in order to fully understand why behavioral intentions were more positive in the face-to-face job interview. Prior research suggests that behavioral intentions are shaped by applicants' dispositional factors, such as Big Five personality dimensions, cognitive ability (Merkulova et al., 2014) and core self-evaluations (McLarty and Whitman, 2016), because people with higher self-beliefs about their abilities to perform well are more likely to form stronger behavioral intentions (Ajzen, 2011). Perceptions and behavioral outcomes are also shaped by factors such as job-relatedness of the selection procedure (Gilliland, 1993) and evaluations of the interviewer (Sears et al., 2013). Further research should examine how and why such personal and situational factors influence applicants' perceptions of the robot-mediated interview in order to get a better grasp of the benefits and shortcomings of this novel type of interview setup.

### Implications for Research

The study points toward new research questions to be answered. More specifically, the finding that the robot-mediated job interview was perceived as less fair calls for additional reflections. We tentatively suggest the following possible explanations. First, the relevant target group for the robot-mediated job interview may be a particular segment of applicants. Applicant perceptions may, for instance, depend on whether they have experienced discrimination. Applicants that have previously experienced discrimination during selection and recruitment processes may perceive the robot-mediated job interview differently (i.e., more positively) than those (with limited job search and work experience) who have felt justly treated and who have the expectations of being “in high demand” on the job market, such as our respondents.

Second, the degree of novelty of the technology and its use in a new context (job interview) may have had an effect on the resulting fairness perceptions. First impressions of a robot are likely to be biased, but they are expected to change during interactions with the robot (Dautenhahn, 2007). Indeed, research suggests that it takes a couple of minutes for subjects to get used to the Telenoid and the novel situation in which the robot is used (Seibt and Vestergaard, 2018). However, our online vignette-based experimental survey only involved very brief video stimuli rather than interaction or engagement with the robot. The respondents' first impressions of the robot therefore did not have a chance to settle and thus remained unchallenged (Smedegaard, 2019).

Third, the finding also prompts considerations related to the design of a robotic fair proxy. While a number of visual cues (e.g., from body gestures to facial expressions, from gender to race) can trigger negative assessments, it is possible that removing only a few specific rather than the entire range of cues would be perceived more positively by applicants. Furthermore, different segments of applicants may prefer to remove different visual cues. The insights from the open-ended question in our survey also indicate that the design of the Telenoid, which was used in the study, may be perceived as unusual and even creepy and/or distresing. While it could be argued that the “minimal design” of the Telenoid may hold advantages for reducing implicit biases, some respondents perceived it as “a doll” and “bizarre,” thus indicating a potential uncanny valley effect (Mori et al., 2012).

Four, the robot-mediated job interview may be perceived as less fair because it places a technology in between the interviewer and the applicant. Such “intervention” may be viewed as a way to control the selection process rather than make it fairer. In addition, compared with face-to-face communication, technology-mediated communication has been shown to increase the degree to which people open up and engage in spontaneous self-disclosure (Joinson, 2001). Similarly, this may elicit negative perceptions of the robot-mediated interview, and be viewed as a way of manipulating the applicant to open up more than she would normally, thus reducing fairness perceptions of the robot-mediated job interview. Future research could examine whether a different framing of the robot-mediated interview situation would lead to different results.

Five, inexperienced jobseekers may perceive (more) useful experiences from engaging in face-to-face communication in their job search. Experiencing the face-to-face interview will have the advantage of learning how interviewers react verbally and non-verbally to different self-presentation and communication strategies of job applicants, which would promote the applicants' learning about how to perform well during job interviews. This experience may be the necessary foundation before an applicant is able to reap the benefits of the robot-mediated job interview.

Lastly, the lower fairness perceptions of the investigated robot-mediated interview may be due to the symmetrical nature of the FPC in our study. While the symmetrical version of the FPC is intended to make the job interview fairer for both interviewers and applicants, it may be perceived as having too great a psychological distance (Trope and Liberman, 2010) between the communicating parties. A job interview is a high-stake context in which applicants want to make a good impression, which demands a certain degree of self-promotion (Schreurs et al., 2018). This degree of self-promotion can be more easily adjusted if the applicant is able to see the non-verbal reactions of the interviewer, which is possible in the asymmetrical version of the FPC. However, in the symmetrical version of the FPC, this is not the case. If applicants consider self-promotion as being an important part of the job interview, then removing the possibility to adjust the self-promotion tactics to the reactions of the interviewer may have a negative effect on their perceptions of the robot-mediated setup. Nonetheless, such possibility for impression management may bias the judgement of the interviewer (Howard and Ferris, 1996). Future research could thus investigate the boundary conditions for the robot-mediated job interview, in particular the relevant target group(s), and how different target groups perceive the symmetrical and asymmetrical versions of the robot-mediated job interview.

### Implications for Practice

The study, has also some practical implications. The design of a robotic agent for personnel selection is not only pertinent because of the way the robot itself may be perceived by applicants, but also because the robot's design is likely to have consequences for the way the organization is perceived. This is because the robot is, in a way, an organizational representative (Nørskov and Ulhøi, 2020), and thus may influence the applicant's perceptions of the organizational attraction (Turban and Dougherty, 1992). This may in turn have consequences for organizational reputation (McCarthy et al., 2017) and its ability to attract applicants (Ryan and Huth, 2008). Consequently, there is a need for hiring organizations and robot designers to consider how the kinetic, physical and functional features of a robot may promote interaction between applicants and interviewers in desirable ways. With respect to the design of the robot used in this study, the responses to the open-ended question in the survey indicated that the robot's physical appearance may have been one of the reasons why the robot-mediated interview was not perceived more positively. On the one hand, this can be related to the respondents' limited exposure time to such a novel concept and technology, as discussed above. On the other hand, it is worthwhile considering whether the robot should be able to transmit more of the facial mimicry and body movements. Additional features of this type would, however, lead to some trade-offs if the “minimalist” design of the Telenoid is changed. Adding features that increase facial mimicry (e.g., smiling and blinking) and body movement (e.g., leaning forward) may lead to even more social presence, and to getting a “better feel” of the applicant. Indeed, recent research reveals that adding more humanlike kinetic cues to a social robot increases the perceived intimacy of the interaction (Xu, 2019). However, adding such features will entail the disadvantage of allowing certain triggers to perceptual biases, as applicants with more lively facial mimicry and body movement are typically associated with being extravert, which may lead to favorability bias and more positive assessments (Paulhus and Morgan, 1997; Swann and Rentfrow, 2001). The design of a robotic fair proxy in job interviews thus needs to be able to strike a delicate balance between allowing enough social cues into the interaction while at the same time reducing the presence of those cues that are most likely to trigger implicit biases and potentially lead to discrimination of applicants. Such design also needs to be aligned with the image that the hiring organization seeks to signal to its external stakeholders regarding the type of workplace the organization is or aims to be.

### Limitations

Several limitations apply to this study. *First*, the use of business students calls for some reservations. Despite the fact that such populations have long been an accepted tradition in social psychology and business studies, they do of course not reflect a representative sample of job applicants and the associated probabilities of experiencing discrimination. We chose students, as they are “born digitals” and thus expected to be used to new technologies. Given that our respondents are young, in high demand after completion of their education, and have limited experience with job interviews, their previous opportunities for experiencing possible discrimination are likely to be low. *Second*, as discussed above, the exposure time to the video-based stimuli material in the experimental survey was relatively short, around 40 s. There is a tradeoff, however, between adding more time-consuming features to an online survey and securing a satisfactory response rate. Allowing for longer exposure to a novel technology in a setup that currently does not use such technology may have offset the respondents' first impressions and conceptions of the robot-mediated job interview and changed them. Third and relatedly, that the respondents were not physically engaged in the interaction may have affected their stance toward the robot. It has been found that the physical presence of a robot affects the extent to which respondents will rate a robot positively and which interactions they can imagine engaging in with said robot (Bainbridge et al., 2011). *Finally*, witnessing an interaction with a robot is likely a new experience to the majority of respondents, which may predispose them to react with surprise or indecision—reactions that may be intensified when taking the third-person perspective (Kahn et al., 2011; Turkle, 2011). The exact nature and impact of these issues on the present study cannot be determined.

## Conclusion

By relying on an online video vignette-based experimental survey, this paper examined how the use of new technology during employment interviews affects applicants' fairness perceptions. Using a robot as a fair proxy in the employment interview is a novel approach for conducting interviews and has as yet not been experienced by applicants. Our findings show that the robot-mediated interview is perceived as less fair than the face-to-face interview. Nonetheless, as limitations of our study indirectly suggest, it would be important to test this interview technique across different segments of applicants to establish whether certain groups in the labor market who, for instance, given their previous experiences with discrimination in job interviews, and their socio-economic and/or socio-cultural background may be more likely to express different perceptions of the robot-mediated and face-to-face interviews.

## Data Availability Statement

The raw data supporting the conclusions of this article will be made available by the authors, without undue reservation.

## Ethics Statement

Written informed consent was obtained from the individuals for the publication of any potentially identifiable images or data included in this article.

## Author Contributions

SN contributed to the conceptualization, research design, data collection, and took the lead in writing the manuscript. MD and JU were involved in conceptualization, research design, data collection, and writing. MJ conducted the data analysis and contributed to the writing of the manuscript. CE was involved in the conceptualization and research design. JS conceived the main idea for the study, and was involved in the conceptualization. All authors contributed to the article and approved the submitted version.

## Conflict of Interest

The authors declare that the research was conducted in the absence of any commercial or financial relationships that could be construed as a potential conflict of interest.
